# Profiling truncated variants of TCTN1 unveils the essential role of its integrity for ciliogenesis

**DOI:** 10.1016/j.isci.2025.114190

**Published:** 2025-11-24

**Authors:** Huimin Yu, Lin Jiang, Chuang Xu, Ya Li, Suhui Wang, Zhao Ma, Yunyao Qian, Yiqiong Wu, Chenyu Miao, Zhouzhou Dong, Liang Wang

**Affiliations:** 1 School of Life Sciences, Jiangsu Normal University, Xuzhou 221116, China

**Keywords:** Natural sciences, Biological sciences, Biochemistry, Cell biology

## Abstract

Cilia protrude from the cell surface to execute signal transduction and cell motility. Tectonic (TCTN) is a transition-zone (TZ) “gatekeeper” that determines ciliary composition and function. The role of its only conserved module in the TCTN protein family, DUF1619, lacks functional annotation. Herein, we quantitatively compared full-length TCTN1 and four truncations (DUF1619, ΔDUF1619, N-DUF1619, and DUF1619-C) stably expressed in *Chlamydomonas reinhardtii tctn1* cells. Surprisingly, neither fragment could rescue ciliary morphology of *tctn1*; DUF1619 alone aggravates ciliary defects. In particular, deletion of either terminus decreases TZ localization and, for the C-terminus, destabilizes the protein during ciliary disassembly. Thus, our findings demonstrate that the full-length TCTN1, rather than DUF1619 alone, is indispensable for ciliary integrity.

## Highlights

• Ciliary integrity requires full-length TCTN1 with conserved DUF1619 domain
• Sole DUF1619 shows cytotoxicity on cellular and ciliary phenotypes
• N/C-tails of TCTN1 contribute to protein stability and transition zone targeting

## Graphical abstract

**Figure:**
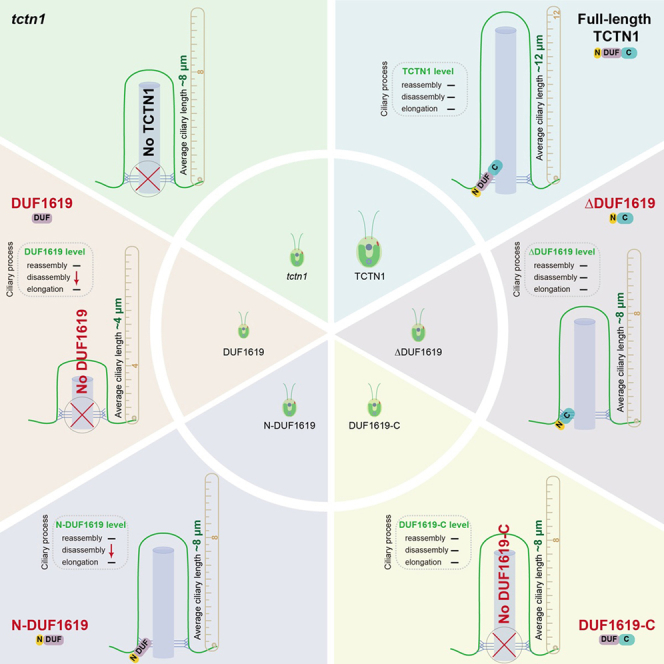

## Introduction

Cilia (also known as flagella) are microtubule-based and highly conserved cellular “antenna” structures that mediate cellular signal transduction and motility through their regulation of numerous ciliary molecules inside cilia.^1^^,^^2^^,^^3^ A specialized ciliary base structure named the ciliary transition zone (TZ), with typically Y-links, functions as a “gatekeeper” to control the specific ciliary composition by governing protein transportation into/out of the cilia.^4^^,^^5^^,^^6^^,^^7^ Therefore, the TZ maintains cellular physiological homeostasis via its regulation at the base. Currently, abnormalities in the TZ lead to ciliary defects, resulting in a broad spectrum of disorders and symptoms, including brain, kidney, liver, eye, bone, and other organs, which typically appear in the form of syndromes termed Meckel-Gruber syndrome (MKS), nephronophthisis (NPHP), Joubert syndrome (JBTS), and oral-facial-digital (OFD) syndrome, collectively designated ciliopathies.^8^^,^^9^^,^^10^^,^^11^^,^^12^ With the advantages of proteomics and superresolution imaging technology, the molecular components and precise localizations of TZ proteins are well characterized and are primarily composed of two TZ complexes, MKSs (MKS1, MKS2, B9D1, B9D2, TCTN, etc.) and NPHPs (NPHP1, NPHP4, NPHP8, etc.), which are organized into 9-fold symmetric rings in which MKSs are generally located more peripherally than NPHPs.^13^^,^^14^^,^^15^^,^^16^ Mutations in TZ genes are among the primary causes of ciliopathy, suggesting their crucial and cooperative roles in cilia.^17^ However, studies on individual TZ genes are still lacking, which hinders the understanding of the molecular mechanisms of ciliopathy caused by every single TZ gene mutation.

Tectonic (TCTN), derived from ancient Greek, means construction, showing its fundamental role in constructing cellular morphology.^18^ This family typically consists of 1–3 members in ciliated model organisms, including membrane-bound proteins and single-transmembrane proteins, and is evolutionarily conserved TZ proteins among different species.^18^^,^^19^^,^^20^ Our group and other research groups have demonstrated that the TCTN protein family is vital as a ciliary gate molecule for ciliogenesis, ciliary composition, hedgehog signaling, and ciliary ectosome formation.^20^^,^^21^^,^^22^^,^^23^^,^^24^ Current clinical case studies show that mutations and variations in each of the three *TCTN* genes in humans cause various ciliopathies by affecting ciliary biogenesis and hedgehog signaling.^8^^,^^24^^,^^25^^,^^26^^,^^27^^,^^28^^,^^29^^,^^30^ Insights into TCTN protein domain architecture revealed that the protein family contains a Cys-rich N-terminal region and a tectonic-specific domain named DUF1619 (Pfam: PF07773; InterPro: IPR011677).^31^^,^^32^ To date, the function and mechanism of this conserved DUF1619 domain in controlling ciliary gating remain ambiguous.

In the present work, to elucidate DUF1619 and the functional segment of the TCTN protein, we designed and transformed different truncated forms of *TCTN1*-expressing plasmids (DUF1619, ΔDUF1619, N-DUF1619, and DUF1619-C) within the genetic background of the *tctn1* cell line, a *TCTN1*-null mutant in *Chlamydomonas reinhardtii* (*C. reinhardtii*). An analysis of various cilia-related processes, including ciliary assembly, reassembly, disassembly, and elongation, revealed that all these truncated TCTN1 proteins fail to rescue the ciliary phenotypes of *tctn1*, indicating that the integrity of TCTN1 is indispensable for its ciliary functions. Unexpectedly, we found that only TCTN1(DUF1619)-expressing cells presented more severe ciliary and cellular morphology, shorter cilia, a lower ciliation ratio, slower ciliary kinetics, and smaller cells. Furthermore, dissectional analysis of the protein level/localization by immunoblotting and immunostaining revealed no dramatic change in the expression or location of TZ molecules (NPHP4 and NPHP8) or ciliary protein (BBS1) in these truncated variants during the above ciliary events. Additionally, deletion of the C-terminal region of TCTN1 attenuated the corresponding protein stability underlying the ciliary disassembly process, and both N- and C-terminal segments of TCTN1 contribute to the ciliary base targeting ability of TCTN1, highlighting the dual roles of the C-terminal region of TCTN1 in maintaining the stability of full-length TCTN1 during ciliary depolymerization and the primarily ciliary TZ targeting signal at its N-terminus. These collective data suggest that the DUF1619 domain, along with the N- and C-terminals of TCTN1, coordinates ciliary biofunctions to maintain the ciliary morphology.

## Results

### Construction and screening of *C. reinhardtii* cell lines expressing various truncated TCTN1 proteins

**Figure 1:**
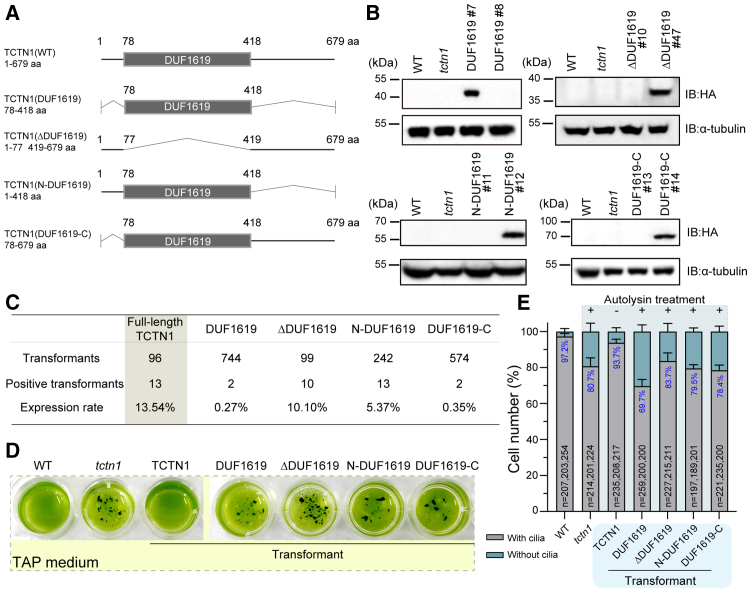
Screening and characterization of *C. reinhardtii* truncated TCTN1 mutants (A) Diagram of the protein structure of TCTN1 with a conserved DUF1619 domain and truncated versions of the TCTN1 protein for dissection. The key sites of TCTN1 (679 aa) are labeled, in which the protein segment at positions 78–418 corresponds to the DUF1619 domain (gray box) with an unknown function. (B) Screening of the indicated transformants expressing the truncated versions of TCTN1 (DUF1619, ΔDUF1619, N-DUF1619, and DUF1619-C) by immunoblotting (IB). WT (wild-type) and *tctn1* cell samples were used as negative controls. Antibodies against HA and α-tubulin were used for screening HA-tagged truncated TCTN1 and as a loading control, respectively. The molecular masses of standard proteins in kDa are indicated. (C) A statistical table summarizing the numbers of screened transformants, positive transformants, and the expression rates of full-length TCTN1 and truncated TCTN1 for various constructs. (D) Phenotypes of WT, *tctn1*, full-length TCTN1 rescued cells (abbreviated as TCTN1) and truncated mutants in a 24-well plate with TAP medium. (E) The number of cells with/without cilia was calculated and plotted with a column graph. Truncated mutants and *tctn1* as control were treated with autolysin (+) for ciliogenesis before the ciliation rate was calculated because of their palmelloid ability. WT and the rescued (TCTN1) cell line were directly counted for their ciliation rates as positive controls. The counted cell numbers (black) and the ciliation rate (blue) were marked on the column under three repeated experiments.

Our previous research demonstrated that TCTN1 in *C. reinhardtii* plays a fundamental role in ciliary gating mechanisms to ensure proper ciliary morphology and ectosome formation.^23^ Full-length TCTN1 (abbreviated as TCTN1) could rescue the *tctn1* cell line, which is a *TCTN1*-null mutant.^23^ To illustrate the ciliary functions of the conserved tectonic domain (DUF1619, Pfam: PF07773; InterPro: IPR011677) in the TCTN protein family and search for a functional segment of TCTN1 in rescue experiments, we constructed four different truncated *TCTN1* plasmids for the expression of corresponding versions of the TCTN1 protein, named TCTN1(DUF1619), TCTN1(ΔDUF1619), TCTN1(N-DUF1619), and TCTN1(DUF1619-C), abbreviated as DUF1619, ΔDUF1619, N-DUF1619, and DUF1619-C, all of which were fused with a 3×HA tag at their C-terminals according to our previous design strategy of the full-length *TCTN1*-expressing plasmid (Figure 1A).^23^ Upon transformation into the *tctn1* cell line, an immunoblotting screening assay was performed by using an HA antibody to isolate any immunoreactive positive transformants with the correct molecular weights from the corresponding transformant library. All four different truncated *TCTN1*-expressing cell lines (DUF1619, ∼39 kDa; ΔDUF1619, ∼39 kDa; N-DUF1619, ∼47 kDa; DUF1619-C, ∼67 kDa) were obtained by immunoblotting screening (Figure 1B). Meanwhile, the single western blot (WB) gel analysis also indicated that the protein amounts of truncated TCTN1 in different strains were very different, among which N-DUF1619 expressed the most, followed by DUF1619, which were at least 10 times more than that of the full-length *TCTN1*-expressing strain (Figure S1). Screening assays for truncated *TCTN1*-expressing cell lines revealed that the expression rate varied among different versions of TCTN1, ranging from 0.27% to 10.10%, compared with 13.54% of full-length one (Figure 1C). To further understand whether these truncated *TCTN1*-expressing cell lines have a rescue function, we analyzed their phenotypes in TAP medium and found that all of them phenocopied the *tctn1* mutant, which formed aggregates in the medium (Figure 1D). Additionally, their ciliation ratio after autolysin treatment was comparable to that in *tctn1* (Figure 1E). These collective data demonstrate that the expression of the intact TCTN1 is critical for the normal ciliary function.

### Noncomplementation of the ciliary phenotype in *tctn1* by different truncated mutants

**Figure 2:**
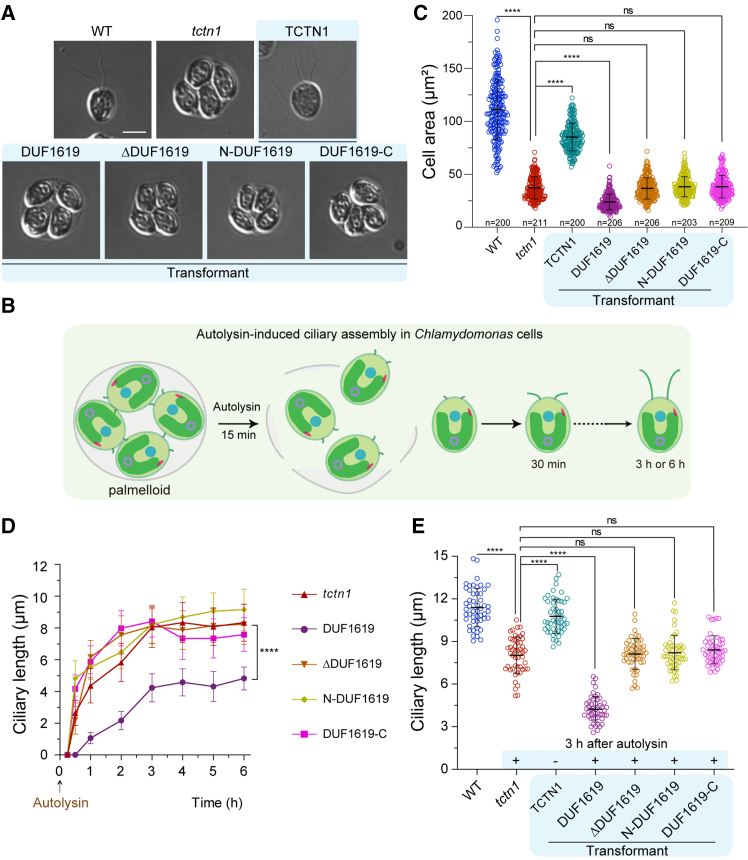
Truncated versions of TCTN1 cannot rescue ciliary defects in the *tctn1* mutant (A) DIC images displaying two normal cilia in front of WT and the full-length TCTN1 rescued (abbreviated as TCTN1) cells and palmelloid cells in *tctn1* and truncated mutant cells. Scale bars, 5 μm (B) Cartoon depicting the autolysin-induced ciliary assembly process in *Chlamydomonas* cells. (C) Scatter graph showing the cell area (μm^2^) among the WT, *tctn1*, TCTN1, and truncated mutants. The mutants, including *tctn1* and truncated cells due to their palmelloid, were first treated with autolysin to release the cells and then used for measurements of the cell area. The numbers of measured cells are indicated. Statistical significance to the *tctn1* group was determined via one-way ANOVA. ns, not significant. ∗∗∗∗*p* < 0.0001. (D) Ciliary assembly curves of *tctn1* and truncated mutant cells after treatment with autolysin were plotted to view their differential assembly kinetics. The data are the means ± SDs (*n* = 50). Statistical significance to the *tctn1* group was determined via two-way ANOVA. ∗∗∗∗*p* < 0.0001. (E) Scatter graph showing distinguished ciliary length in *tctn1* and truncated mutant cells. The mutants, including *tctn1* and truncated cells due to their palmelloid phenotype, were treated with autolysin (+) for 3 h for ciliogenesis and then for measurements of ciliary length. The data are presented as the means ± SDs (*n* = 50). Statistical significance to the *tctn1* group was determined via one-way ANOVA. ns, not significant. ∗∗∗∗*p* < 0.0001.

To further reveal the microscopic cell phenotypes of these truncated mutants, differential interference contrast (DIC) images revealed that *tctn1* and other truncated mutants were palmelloid and immotile in TAP medium, while the TCTN1 rescued cell line was morphologically identical to the WT (wild-type) strain (Figure 2A). Autolysin, a protease commonly used to lyse cell walls, is employed to degrade the cell wall and free daughter cells for ciliation (Figure 2B).^33^ After treatment with autolysin and replacing the medium with fresh medium, all daughter cells simultaneously elongated their cilia. All the cells were subsequently subjected to analysis of the cell area, ciliary regeneration process, and ciliary length. Compared with those of WT, TCTN1, and *tctn1*, the cell areas of the truncated cell line were similar to those of *tctn1* (∼38 μm^2^), but the DUF1619 cell line had a severely smaller cell area, at only 23.93 ± 7.34 μm^2^ (Figure 2C). Similarly, during the autolysin-induced ciliary assembly process, the DUF1619 truncation mutant presented a slower regeneration rate (∼0.8 μm/h) within the 6 h than did the other mutants and *tctn1* cells (1.3–1.5 μm/h) (Figure 2D). The final ciliary lengths of these truncated mutants were comparable to those of *tctn1*, except for the DUF1619 mutant, which presented shorter cilia (4.23 ± 0.89 μm) (Figure 2E). These results illustrate that the complementary expression of the DUF1619 domain alone is invalid and even crosscurrent.

### Severely defective ciliary phenotype in the DUF1619-expressing cell line

**Figure 3:**
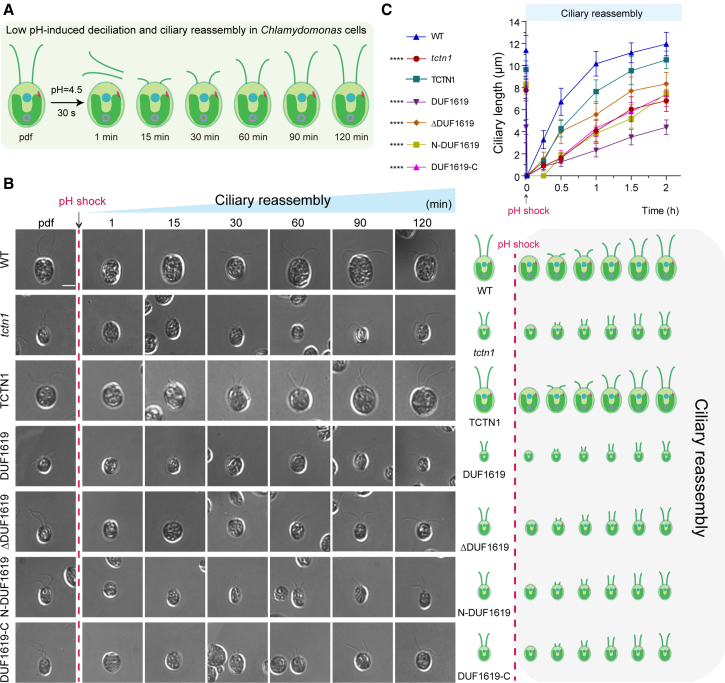
The TCTN1(DUF1619) cell line shows severe ciliary defects in ciliary reassembly (A) Cartoon depicting the low pH (pH shock)-induced deciliation and ciliary reassembly process in *Chlamydomonas* cells. (B) DIC images displaying pH-shock-induced deciliation and ciliary reassembly in WT, *tctn1*, TCTN1, and truncated mutant cells. The black arrow indicates the time of pH shock. The sampling time is timed with the deciliation time as the starting point. pdf, predeflagellated. Scale bars, 5 μm. (C) Ciliary reassembly curves after pH shock in WT, *tctn1*, TCTN1, and truncated mutant cells were plotted to view their differential reassembly kinetics. The data are presented as the means ± SDs (*n* = 50). Statistical significance to the WT group was determined via two-way ANOVA. ∗∗∗∗*p* < 0.0001. See also Figures S2 and S3 for ciliary disassembly and elongation processes.

The ciliary base compartment, named the ciliary transition zone, is believed to constitute the ciliary gate for the regulation of ciliary reassembly, disassembly, and elongation processes.^4^ Our previous data demonstrated that ciliary basal TCTN1 is crucial for ciliary phenotypes.^23^ The above results revealed that DUF1619 mutant cells were extremely small with short cilia. To further elucidate the ciliary phenotypes of the DUF1619-expressing cell line during various ciliary processes, we performed low-pH-shock-induced ciliary reassembly, NaPPi-induced ciliary disassembly, and LiCl-induced ciliary elongation in the DUF1619 mutant and other cell lines (Figures 3A, S2A, and S3A). DIC images during the ciliary reassembly process and corresponding plots confirmed that all cells from the WT, TCTN1, *tctn1*, and various truncated mutants could reassemble cilia to their respective original lengths within 2 h after pH shock; however, the DUF1619 mutant regenerated poorly (Figures 3B and 3C). Similarly, during the ciliary disassembly, the DUF1619 mutant exhibited faster ciliary disassembly within 2 h due to its shorter cilia, whereas other cell lines needed at least 3 h to disassemble their original cilia (Figures S2B and S2C). During ciliary elongation, the DUF1619 mutant exhibited an impotent elongation response to LiCl, resulting in a final ciliary length of approximately 5 μm (Figures S3B and S3C). Thus, the DUF1619 mutant is an impotent cell line for various ciliary processes.

### Reduced TCTN1 protein stability due to the absence of its C-terminus during ciliary disassembly

**Figure 4:**
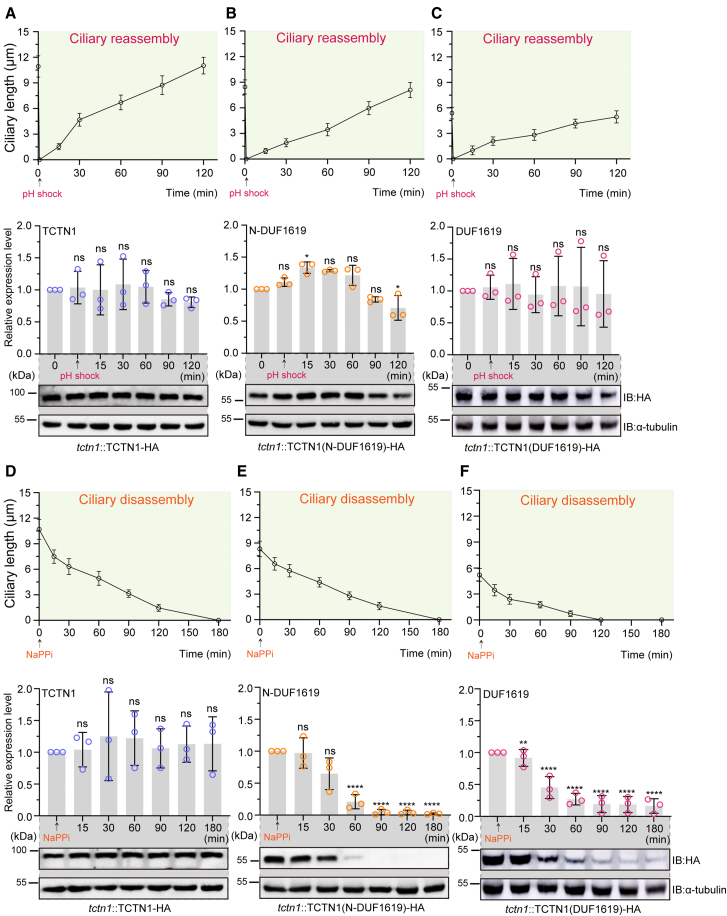
The C-terminus of TCTN1 stabilizes TCTN1 during ciliary disassembly (A–C) Ciliary reassembly graphs and the corresponding immunoblot analysis, along with band intensity by gray values in *tctn1*::TCTN1-HA (A), *tctn1*::TCTN1(N-DUF1619)-HA (B), and *tctn1*::TCTN1(DUF1619)-HA (C) cells, were plotted to view the changes in the corresponding protein levels. (D–F) Ciliary disassembly graphs and the corresponding immunoblot analysis data, along with band intensities by gray values in *tctn1*::TCTN1-HA (D), *tctn1*::TCTN1(N-DUF1619)-HA (E), and *tctn1*::TCTN1(DUF1619)-HA (F) cells, were plotted to view the changes in the corresponding protein levels. The data in the ciliary length curves are the means ± SDs (*n* = 50). The data at the relative protein level are presented as the means ± SDs (*n* = 3). Statistical significance to the time point 0 group was determined via one-way ANOVA. ns, not significant. ∗*p* < 0.1; ∗∗*p* < 0.01; ∗∗∗∗*p* < 0.0001. The molecular masses of standard proteins in kDa are indicated. α-tubulin was used as a loading control. See also Figure S4 for the ciliary elongation process and Figures S5 and S6 for TCTN1(ΔDUF1619) and TCTN1(DUF1619-C) strains in various ciliary events.

Ciliary dynamics, including regeneration, shortening, and elongation, involve intraflagellar transport complexes and other cargos composed of “vehicles” passing through the regulatory TZ between the cilia and cytoplasm to maintain the ciliary dynamics.^5^^,^^34^ In order to reveal whether the ciliary gating function of TZ is regulated by the protein level changes of TZ molecules, we investigated the expression profiles of these truncated *TCTN1* genes during various ciliary processes. During the different ciliary dynamic processes, all strains synchronously reassembled, disassembled, or elongated their respective cilia, and the corresponding versions of TCTN1 with a 3×HA tag at different indicated time points were analyzed via WB against the HA tag. Interestingly, compared with those in the full-length *TCTN1*-expressing cell line (TCTN1), the levels of the protein TCTN1 lacking the C-terminal domain, including N-DUF1619 and DUF1619, were consistent in the process of ciliary assembly and elongation (Figures 4A–4C and S4) but gradually decreased during ciliary disassembly (Figures 4D–4F). For ΔDUF1619 and DUF1619-C, the versions of TCTN1 containing the C-terminal domain exhibited relative stability during all tested ciliary dynamic processes (Figures S5 and S6). Collectively, the abovementioned results indicate that the C-terminus of TCTN1 plays a vital role in its stability during the ciliary shortening.

### The ciliary base localization sequences at the N- and C-termini of TCTN1

**Figure 5:**
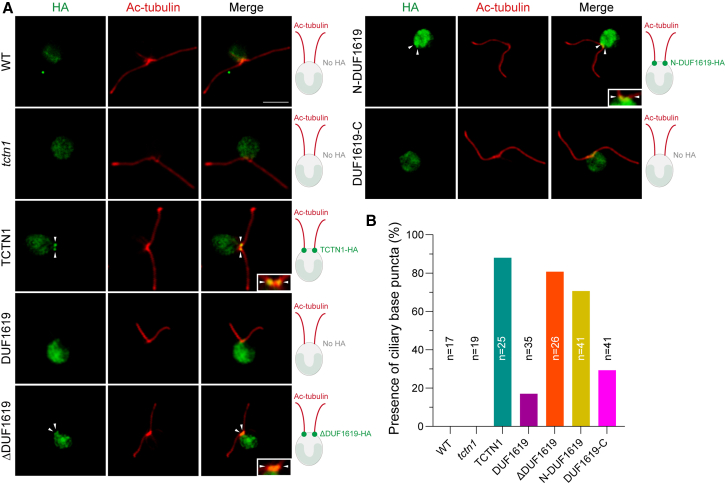
The N- and C-termini dependency for TCTN1 in the transition zone localization (A) Immunostaining images showing the ciliary base signals using HA antibody (green) in WT, *tctn1*, TCTN1, DUF1619, ΔDUF1619, N-DUF1619, and DUF1619-C cell lines. The signal of acetylated α-tubulin (Ac-tubulin, red) marks the cilium. The white arrowheads mark the ciliary base (ciliary transition zone). The insets show higher magnification views of the transition zone puncta. The statistical analysis of the percentage of fluorescence localization at the TZ is also presented (*n* = 17, 19, 25, 35, 26, 41, and 41). Scale bars, 5 μm.

All the abovementioned data showed that all truncated TCTN1 could not phenologically rescue the *tctn1* cell line. To determine whether the loss of TZ localization contributes to the failure of phenotypic rescue in the *tctn1* cell line and which segment of TCTN1 is responsible for the ciliary base localization, an immunofluorescence (IF) assay using an HA antibody against truncated TCTN1-HA was conducted in the corresponding strains. Notably, the full-length TCTN1 rescued cell line exhibited two prominent puncta at the ciliary base or TZ region (∼88% of cells showing ciliary base signals), which were not observed in the WT and *tctn1* mutant cells (Figure 5), consistent with our previous report.^23^ In contrast, TCTN1 lacking the N-terminus (DUF1619 and DUF1619-C) resulted in a significantly reduced proportion of its TZ localization signal to ∼17% and ∼30%, respectively. Conversely, the percentage of positive ciliary base targeting cells in ΔDUF1619 and N-DUF1619 cells was ∼80% and ∼70%, respectively (Figure 5). These intriguing observations suggest that the ciliary base targeting sequence of TCTN1 primarily resides in the N-terminus and secondarily at its C-terminus. Therefore, the C-terminal region of TCTN1 is essential not only for protein stability but also for ciliary TZ targeting.

### No dramatic changes in other ciliary components underlying truncated TCTN1 cell lines

**Figure 6:**
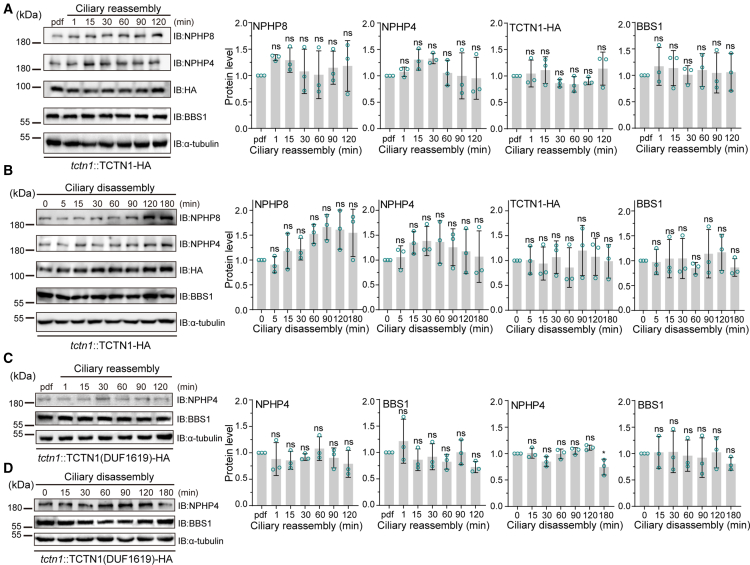
The levels of ciliary proteins are almost unchanged in TCTN1(DUF1619) mutants (A and B) Immunoblot and gray value profiling for changes in the levels of ciliary proteins (NPHP8, NPHP4, BBS1, and TCTN1-HA) during ciliary reassembly (A) and disassembly (B) events in *tctn1*::TCTN1-HA. (C and D) Immunoblot and gray value profiling for changes in the levels of ciliary proteins (NPHP4 and BBS1) during ciliary reassembly (C) and disassembly (D) events in *tctn1*::TCTN1(DUF1619)-HA. pdf, predeflagellated. The sampling times are as indicated in this figure. The data at the relative protein level are presented as the means ± SDs (*n* = 3). Statistical significance to the time point 0 or pdf group was determined via one-way ANOVA. ns, not significant. The molecular masses of standard proteins in kDa are indicated. α-tubulin was used as a loading control. See also Figure S8 for other truncated strains.

**Figure 7:**
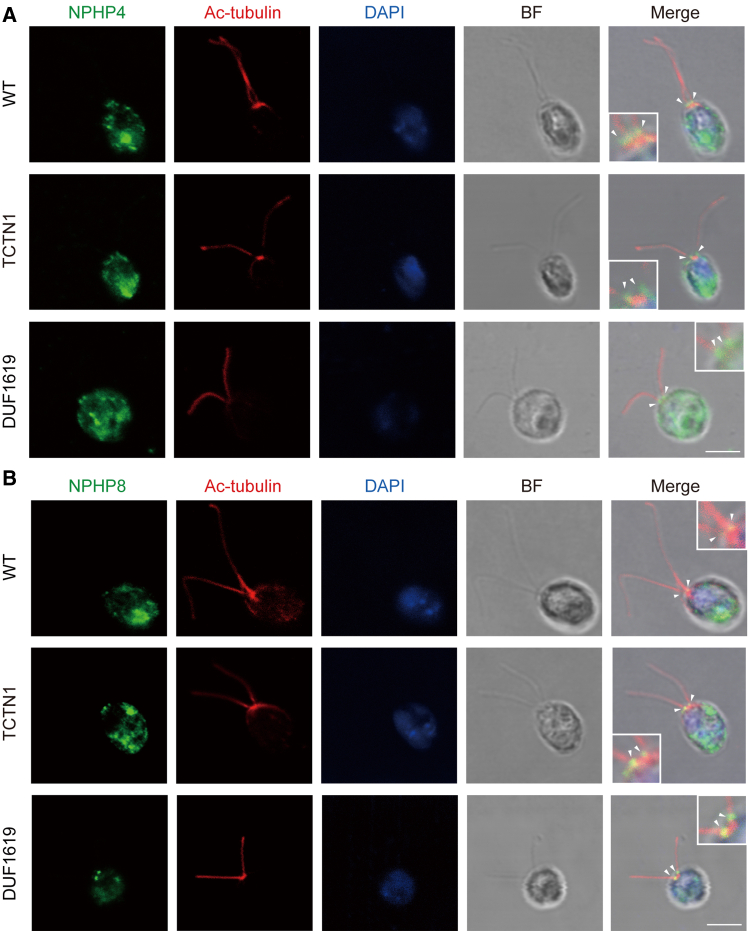
The transition zone localizations of NPHP4 or NPHP8 in truncated TCTN1 mutants are not altered (A and B) Immunostaining images displaying the ciliary base puncta of NPHP4 (A) or NPHP8 (B) in WT, *tctn1*::TCTN1-HA (TCTN1), and *tctn1*::TCTN1(DUF1619)-HA (DUF1619) cells. All the cells were immunostained with anti-NPHP4 (green) or anti-NPHP8 (green) and anti-acetylated α-tubulin (Ac-tubulin, red) antibodies. The nuclei were stained with DAPI (blue). The white arrowheads indicate the ciliary base (ciliary transition zone). The insets show higher magnification views of the transition zone region. The brightfield (BF) and merge channels are also showed. Scale bars, 5 μm. See also Figure S9 for other truncated strains.

TZ complexes are located at the ciliary base and coordinate with the sorting of ciliary components into or out of the cilia.^35^^,^^36^^,^^37^ TCTN1, as the core component of TZ complexes, was demonstrated to be one of the key molecules involved in constructing the TZ ultrastructure in our previous study.^23^ Since the gating molecule TCTN1 needs to form TZ complexes with other gating proteins and, in turn, regulate ciliary structure and function, we profiled the localizations and expression levels of the TZ proteins NPHP8 and NPHP4 or the ciliary transportation protein BBS1 in the genetic background of various truncated TCTN1 mutants. The homemade antibodies against NPHP4, NPHP8, and BBS1 were first validated via WB and immunofluorescence in the WT and corresponding mutants. The specific bands were well recognized by the respective antibodies (Figures S7A–S7C). Similarly, two puncta at the ciliary base were labeled in the WT cells but not in the corresponding mutant cells (Figures S7D and S7E). All three homemade antibodies were subsequently used for either WB or IF. At the indicated times during ciliary reassembly or disassembly, we profiled the levels of NPHP8, NPHP4, and BBS1 in the rescued strain (TCTN1) and the truncated mutants (DUF1619, ΔDUF1619, N-DUF1619, and DUF1619-C). The WB results and the corresponding band intensity profiling revealed that the levels of the TZ protein NPHP8 or NPHP4 and the ciliary transportation protein BBS1 were almost unaltered during both ciliary reassembly and disassembly, although the level of NPHP8 was slightly upregulated (∼1.5-folds) at the end of ciliary disassembly in the rescued strain (Figures 6 and S8). Consistently, IF images revealed two puncta at the ciliary base in the WT, rescued, and various truncated mutant strains (Figures 7 and S9). These data suggested that the DUF1619 may not be responsible for the more severe cellular defects caused by the level changes during ciliary reassembly or disassembly or localizations of the above ciliary components.

## Discussion

The ciliary base compartment TZ, as a vital regulatory region of ciliary morphological construction and signal transduction, has been widely studied as the focus of ciliary research.^4^^,^^5^^,^^22^ Taken together, the evidence shows that the fine-orchestration and fine-tuned regulation of TZ protein complexes in this region are indispensable for maintaining the functions of TZ and involve functional domains of various TZ molecules that mediate cellular localizations, interactions, and modifications. However, the lack of studies described above has limited our understanding of how the TZ complexes comprehensively control ciliary composition and function. In this manuscript, our comprehensive analysis of a key molecule, TCTN1, and its truncated variants elucidates the critical role of its structural integrity in ciliary TZ function underlying various ciliary events, especially that the C-terminus of TCTN1 is responsible for its TZ location and protein stability and its N-terminus for the primary ciliary base targeting domain.

As the specific domain found in the TCTN protein family, the DUF1619 domain in ciliated organisms is generally ∼200–350 aa in length and is rich in cysteine.^13^^,^^19^^,^^31^^,^^38^ However, the role of DUF1619 in the control of ciliogenesis and homeostasis remains ambiguous. In the human RPE1 cell line, deletion of this domain in TCTN2 does not effectively rescue it by recruiting the ciliary membrane proteins ARL13B and TMEM67; in the same way, the loss of the N-terminal and C-terminal segments in TCTN2 also cannot restore the enrichment of their ciliary membrane proteins, reflecting the importance of the integrity of TCTN2 in this model organism.^31^ In that study, however, we do not know whether the different truncated variants in the *TCTN2*^−/−^ mutant could rescue the ciliary phenotypes, such as the ciliary ratio. Moreover, from their reported immunofluorescence images, it seems that loss of TCTN2 does not affect ciliary morphology, including the ciliary length, and different variants of TCTN2 phenocopy the *TCTN2*^−/−^ mutant,^31^ which is quite different from our findings. One possible reason is that multiple TCTN members exist in humans with certain functional redundancy. Consistent with the deletion variants of TCTN2 in humans, our results concerning the truncated variants of TCTN1 in *C. reinhardtii* also indicate that the integrity of TCTN1 is required for its ciliary function (Figures 1 and 2). Surprisingly, when the truncated TCTN1 segment containing only the DUF1619 domain is expressed in *tctn1* mutant cells, this mutant presents more serious cellular and ciliary defects, including a reduced cellular area, decreased ciliation rate, shorter length of cilia, and weaker responses in ciliary kinetics, than other types of variants do (Figures 1, 2, 3, S2 and S3). Although the protein levels of different forms of TCTN1 are completely different, a small amount of TCTN1 is sufficient to rescue the *tctn1* mutant (Figures 1 and S1), indicating that the low levels of truncated TCTN1 in the various strains are not the reason for the failure to rescue. Our goal was to search for the minimum functional TCTN1 protein segment to effectively rescue the *TCTN1* mutant in hope of obtaining relevant genetic information about TCTN1 for future gene therapy. However, our data suggest that the DUF1619 domain should coordinate with other necessary motifs or domains to fulfill its functional role. One possibility may be that the cellular functions of the specific DUF1619 domain in this TCTN protein family depend on the complete protein sequence to form the spatial structure or the effective binding of interactive proteins. For example, IFT74 coordinates with IFT81 to form a tubulin-binding complex independent of its N-terminal first 40 amino acids, while its N-terminal first 40 amino acids are responsible for tubulin binding and transportation,^39^^,^^40^ suggesting that the combination of different motifs/domains facilitates protein functions.

Our data also indicate that the expression of the DUF1619 domain alone is detrimental to the cell and that this version of TCTN1 may recruit other unrelated proteins to further disrupt ciliary function, resulting in more severe defects. Moreover, this type of cytotoxicity does not appear to be associated with the overexpression of DUF1619, as the N-DUF1619 strain exhibits a higher level of N-DUF1619 but maintains a phenotype consistent with *tctn1*. Unlike comprehensive complementary studies of the key TZ protein CEP290, the C-terminal domain of CEP290 could effectively rescue retinal degeneration in the *CEP290* mutant as a promising gene therapy strategy,^41^ while the expression of N-terminal CEP290 could also restore the interactor’s TZ localization and ameliorate defects in TZ initiation in the corresponding mutant.^42^ Therefore, seeking the minimum functional domain of this protein family may be futile, or removing the nonfunctional TCTN1 protein segment is extremely limited. To date, functional information on DUF1619 in the TCTN family is still limited. In future research directions, 3D structure prediction assisted by the AlphaFold server and the interactomes of the TCTN complex should be addressed to obtain more physiological information about TCTN proteins.

In addition, the stability of TCTN1 in *C. reinhardtii* is significantly reduced during ciliary disassembly, not during other ciliary processes, when its C-terminus is absent (Figures 4E and 4F), whereas such an event does not occur in the full-length TCTN1-rescued cell line or other truncated *TCTN1*-expressing cells. Furthermore, the N- and C-terminal regions of TCTN1 also harbor two primary ciliary targeting sequences (CTS) to mediate its effective localization to the base of cilia (Figure 5), although this study did not carefully dissect which specific amino acids are mainly responsible for the TZ targeting. These findings indicate that the 261 amino acids in the C-terminus of TCTN1 in *C. reinhardtii* serve at least a dual role in targeting TZ and stabilizing the TCTN1 protein during ciliary disassembly, but not in the processes of ciliary reassembly and elongation. Previous studies have demonstrated that the CTS of many ciliary proteins are located at either the N-terminus or the C-terminus of the proteins, and these sequences exhibit considerable diversity and complexity.^43^^,^^44^^,^^45^ For example, INPP5E contains multiple CTSs (CTS1-CTS4), which are distributed from the middle region to the C-terminus of this protein.^46^ Similarly, Arl13b requires not only the RVEP motif at the C-terminal region for its primary ciliary localization but also its N-terminal lipid-anchor region and middle coiled-coil region to enhance its ciliary localization.^47^ These examples highlight the complexity of ciliary localization signals and may involve the protein spatial structures, bridging of adaptors, transport, and anchoring within the cilia to ensure their effective and proper localization.

During the fast dynamic phases, including the ciliary assembly/disassembly, ciliary proteins undergo rapid turnover, involving protein synthesis and degradation.^48^^,^^49^ Collective evidence implies that the two major protein degradation systems, the ubiquitin-proteasome system and the autophagy machinery, facilitate the ciliary homeostasis.^50^^,^^51^ NPHP5 (nephrocystin 5), a ciliopathy gene product associated with Senior-Løken syndrome, is ubiquitinated by the E3 ubiquitin ligase and consequently allows ciliary disassembly. Moreover, the deubiquitinase USP9X counteracts the deubiquitylation of NPHP5 and prevents its degradation and ciliary disassembly.^52^ Similarly, in addition to protein synthesis to provide ciliary components, the degradation system also promotes ciliogenesis via the removal of ciliary assembly inhibitors; for example, the ubiquitin-dependent proteolysis of KIF2C induces the assembly of the primary cilium.^53^ Thus, regulating the dynamic stability of cilia-related proteins is crucial for maintaining the morphology and physiology of cilia. Intriguingly, as one of the core components of the TZ complex, RPGRIP1L (also named NPHP8) plays multiple roles in proteasomal activity, autophagic activity, and TZ sorting.^54^^,^^55^ While our model shows that the missing C-terminus of TCTN1 results in loss of protein stability during ciliary disassembly events, investigating whether the stability of TCTN1 is proteasome-mediated or autophagy-mediated, especially whether the TZ complex itself regulates its stability, which will further expand the understanding of the synergistic regulation of the TZ complex, is highly interesting.

### Limitations of the study

Herein, we demonstrate that full-length TCTN1 in *C. reinhardtii* is critical for its normal functions in the cilium and imply that different protein motifs/domains are responsible for various necessary functions. Our study on the integrity of TCTN1 still has several limitations. First, at present, predictive and experimental data concerning the TCTN protein family are lacking. There is only one unknown specific DUF1619 domain in this family. Our studies on the truncated mutants solely rely on this limited information. Future research should focus on the different cellular functions mediated by different TCTN1 protein segments, such as cellular transportation, ciliary localization, and the TZ protein interaction network, to dissect the specific functions of different combinations of amino acids of TCTN1. Second, our results were completely obtained via the *Chlamydomonas* model; although it is a significant ciliary research model, other ciliary species exist with 1–3 TCTN family members, especially in model organisms of humans and mice with three members, and the same motif/domain in different TCTN members may be responsible for various functions. Therefore, we need to systematically study the functions of various motifs/domains of TCTN members in different ciliary model organisms to gain insight into how these family members individually and coordinately function in ciliary events.

## Resource availability

### Lead contact

Further information and requests for resources and reagents should be directed to and will be fulfilled by the lead contact, Liang Wang (wangliang@jsnu.edu.cn).

### Materials availability

*C. reinhardtii* strains and plasmids generated in this study will be provided upon reasonable request from the lead contact, Liang Wang (wangliang@jsnu.edu.cn).

### Data and code availability

• All original western blot and microscopy images and GraphPad files reported in this paper have been deposited at Mendeley Data (https://data.mendeley.com/) and publicly available as of the date of publication. The accession number is listed in the key resources table.
• This paper does not report original code.
• Any additional information required to reanalyze the data reported in this paper is available from the lead contact upon request.

## Acknowledgments

We are grateful to the Analyzing & Test Center (Jiangsu Normal University, China) for their technical support. This work was funded by Basic Research Program of Jiangsu (grant no. BK20231351), the Key Research and Development Program of Xuzhou (grant no. KC23300), and the Priority Academic Program Development of Jiangsu Higher Education Institutions (PAPD). We are also thankful to American Journal Experts (AJE) for the English polishing of this manuscript.

## Author contributions

Conceptualization, H.Y., L.J., and L.W.; methodology, H.Y., L.J., C.X., Y.L., S.W., and Z.M.; validation, C.X., Y.L., S.W., Z.M., Y.Q., Y.W., C.M., and Z.D.; investigation, H.Y., L.J., Y.Q., Y.W., C.M., and Z.D.; formal analysis, H.Y., L.J., and L.W.; writing—original draft, H.Y., L.J., and L.W.; writing—review & editing, L.J. and L.W.; funding acquisition, L.W.; supervision, L.W.

## Declaration of interests

The authors declare no competing interests.

## STAR★Methods

### Key resources table

**Table:** 

REAGENT or RESOURCE	SOURCE	IDENTIFIER
**Antibodies**		

anti-HA high affinity	Roche	Cat#11867423001; RRID: AB_390918
anti-α-tubulin	Proteintech	Cat#11224-1-AP; RRID: AB_2210206
anti-acetylated α-tubulin	Sigma-Aldrich	Cat#T7451; RRID: AB_609894
anti-NPHP4	ABclonal	Custom-made
anti-NPHP8	ABclonal	Custom-made
anti-BBS1	ABclonal	Custom-made
HRP-Goat-Anti-Mouse IgG	Jackson	Cat#115-035-003; RRID: AB_10015289
HRP-Goat-Anti-Rabbit IgG	Jackson	Cat#111-035-003; RRID: AB_2313567
HRP-Goat-Anti-Rat IgG	Jackson	Cat#112-035-003; RRID: AB_2338128
Goat Anti-Mouse IgG H&L (Alexa Fluor® 594) preadsorbed	Abcam	Cat#ab150120; RRID: AB_2631447
Goat Anti-Rat IgG H&L (Alexa Fluor® 488)	Abcam	Cat#ab150157; RRID: AB_2722511
Goat Anti-Rabbit IgG H&L (Alexa Fluor® 488)	Abcam	Cat#ab150077; RRID: AB_2630356

**Bacterial strains**		

Trans5α Chemically Competent Cell	TransGen Biotech	Cat#CD201

**Chemicals, peptides, and recombinant proteins**		

Hygromycin B Solution	Sangon Biotech	Cat#B540725
Lithium chloride	Sigma-Aldrich	Cat#V900067
Sodium pyrophosphate tetra-basic	Sigma-Aldrich	Cat#V900752
cOmplete^TM^ Mini EDTA-free Protease Inhibitor Cocktail	Roche	Cat#11836170001
DAPI-Fluoromount-G	SouthernBiotech	Cat#0100-20
Immunostaining Blocking/Primary Antibody Dilution Solution	Sangon Biotech	Cat#E674004
Immunostaining Secondary Antibody Dilution Buffer	Sangon Biotech	Cat#E674005
Goat Serum	Sangon Biotech	Cat#E510009
Ready-to-Use Seamless Cloning Kit	Sangon Biotech	Cat#B632219
KMLPRTANLEAGAIG-C and C-QAQIKMPMSEPEEVE peptides of BBS1 used for polyclonal antibody preparation	ABclonal	Custom-made
PVSDYGLAADTRDQ-C and C-RSRYGAETELSLE peptides of NPHP8 used for polyclonal antibody preparation	ABclonal	Custom-made
EEIFPPPEINEVDPSAREPPPQVLDF DGPPLERVPSMDMLPRNFSRHSM GAGAGFPPELQELTIAMQRQLELL TRAVDELKDERKSMRNSFIALADRP PPDDYGANAAAQVQRYYDAQRAK RASMQPTPEELQRFLDMEPTRNFG DTSAFSGAQAPDGKLPGTAPSRVL LQQLHDAGMIDALPADVQAILRRK GPPRGPS peptide of NPHP4 used for polyclonal antibody preparation	ABclonal	Custom-made

**Critical commercial assays**		

SanPrep Column DNA Gel Extraction Kit	Sangon Biotech	Cat#B518131
SanPrep Column Plasmid Mini-Preps Kit	Sangon Biotech	Cat#B518191
ECL Enhanced Kit	ABclonal	Cat#RM00021

**Deposited data**		

Original WB, IF, and GraphPad data	This paper	Mendeley Data, V1, doi: https://doi.org/10.17632/dxpzpfgrpr.1

**Experimental models: Organisms/Strains**		

*C. reinhardtii* strain: 21*gr* (mt+)	*Chlamydomonas* Resource Center	CC-1690
*C. reinhardtii* strain: 6145c (mt-)	*Chlamydomonas* Resource Center	CC-2895
*C. reinhardtii* strain: *nphp4* (mt+)	*Chlamydomonas* Resource Center	CC-5113
*C. reinhardtii* strain: *nphp8* (mt-)	*Chlamydomonas* Resource Center	LMJ.RY0402.180475
*C. reinhardtii* strain: *bbs1* (mt-)	*Chlamydomonas* Resource Center	CC-4371
*C. reinhardtii* strain: *tctn1* (mt+)	Liang Wang et al.^23^	–
*C. reinhardtii* strain: *tctn1*::TCTN1-HA (mt+)	Liang Wang et al.^23^	–
*C. reinhardtii* strain: *tctn1*::TCTN1 (DUF1619)-HA (mt+)	This paper	–
*C. reinhardtii* strain: *tctn1*::TCTN1 (ΔDUF1619)-HA (mt+)	This paper	–
*C. reinhardtii* strain: *tctn1*::TCTN1 (N-DUF1619)-HA (mt+)	This paper	–
*C. reinhardtii* strain: *tctn1*::TCTN1 (DUF1619-C)-HA (mt+)	This paper	–

**Oligonucleotides**		

Primers for constructions of truncated *TCTN1*-expressing plasmids, see Table S1	This paper	–

**Recombinant DNA**		

Plasmid: pHyg-3HA	Liang Wang et al.^23^	–
Plasmid: pHyg-gTCTN1-3HA	Liang Wang et al.^23^	–
Plasmid: pHyg-gTCTN1(1–77 + 419-679)-3HA	This paper	–
Plasmid: pHyg-gTCTN1(78–418)-3HA	This paper	–
Plasmid: pHyg-gTCTN1(1–418)-3HA	This paper	–
Plasmid: pHyg-gTCTN1(78–679)-3HA	This paper	–

**Software and algorithms**		

ImageJ software	National Institutes of Health	https://imagej.nih.gov/ij/
GraphPad Prism	GraphPad Software	http://www.graphpad.com/scientific-software/prism/
Adobe Photoshop	Adobe	https://helpx.adobe.com/cn/download-install.html
Adobe Illustrator	Adobe	https://helpx.adobe.com/cn/download-install.html

### Experimental model and study participant details

The wild-type (WT) *C. reinhardtii* strain 21*gr* (CC-1690, mt+) and 6145c (CC-2895, mt-) and the mutant strains *nphp4* (CC-5113, mt+), *nphp8* (LMJ.RY0402.180475, mt-), and *bbs1* (CC-4371, mt-) were originally acquired from the *Chlamydomonas* Resource Center (https://www.chlamycollection.org) at the University of Minnesota, USA. The obtained strain was stored in liquid nitrogen using a cell storage buffer (3% methanol in TAP medium) for long-term preservation. The transition zone gene *TCTN1* reported in our previous work was inactivated via insertional mutagenesis with the paromomycin-resistant DNA fragment *aphVIII* in 21*gr*. Generally, the strains were grown on Tris-acetate-phosphate (TAP) plates or in liquid TAP medium with aeration at 23 ± 0.5°C under 8000 lx light intensity with a 14/10 h light/dark cycle. The *tctn1* strain was cultured in TAP liquid medium under continuous lighting conditions for transformation with the indicated linearized plasmids for gene expression. Home-made autolysin from the mating process (mixing 21*gr* & 6145c gametes) was used for 15–20 min of treatment with cell aggregates to free cells for ciliation.

### Method details

#### Plasmid construction, transformation, and screening

The gene expression vector pHyg-3HA harboring hygromycin resistance was described in our previous paper and used for all truncated *TCTN1* expressed in *C. reinhardtii* cells.^23^ The full-length *TCTN1* gene was successfully expressed in the *tctn1* strain via this pHyg-3HA vector, as reported in our previous study.^23^ In general, the pHyg-3HA vector was digested with the restriction endonuclease *Eco*R V, and the inserted DNA fragments, including 1–77 aa and 419–679 aa, were amplified from the template of a full-length *TCTN1*-expressing pHyg-gTCTN1-3HA plasmid via the DJ-F/DJ-1-R1 and DJ-1-F1/DJ-1-R primers via overlap extension PCR. A seamless cloning assay was then applied for assembly of the above-linearized vector and inserts, which subsequently created the final plasmid pHyg-gTCTN1(1–77 + 419-679)-3HA for the expression of TCTN1(ΔDUF1619). The same assembly strategy was used with the DJ-F/DJ-B-R1 and DJ-B-F1/DJ-B-R primers for the construction of the pHyg-gTCTN1(78–418)-3HA plasmid for the expression of TCTN1(DUF1619). For the construction of the plasmid pHyg-gTCTN1(1–418)-3HA expressing TCTN1(N-DUF1619), a 6.5 kb DNA fragment cut by *Cla* I from the plasmid pHyg-gTCTN1(78–418)-3HA as a vector and a 2.3 kb DNA fragment cut by *Cla* I from the plasmid pHyg-gTCTN1-3HA as an insert were ligated by T4 DNA ligase, after which the final construct was obtained. To delete the N-terminus of *TCTN1* for the construction of pHyg-gTCTN1(78–679)-3HA expressing the protein TCTN1(DUF1619-C), the DNA fragment without the N-terminus of *TCTN1* was amplified from the template pHyg-gTCTN1-3HA with primers I124-F2/DJ-B-R1 and DJ-B-F1/I124-R1 via overlap extension PCR and then digested by *Cla* I as the insert. The plasmid pHyg-gTCTN1-3HA was also cut with *Cla* I and purified for an 8.2 kb DNA fragment as a linearized vector. Ligation of the above two *Cla* I-modified fragments was then performed via T4 DNA ligase, and the final plasmid pHyg-gTCTN1(78–679)-3HA was obtained. All primers used to construct the truncated *TCTN1*-expressing plasmids are listed in Table S1. All the above DNA fragments acquired via PCR methods were sequenced and confirmed.

The DNA transformation method was described in detail elsewhere.^56^ All the resulting constructs were linearized with *Nde* I and transformed with 500 ng into *tctn1* cells with a BTX ECM830 electroporator (BTX, USA). Transformants were plated onto hygromycin (Cat. No. B540725, Sangon Biotech, China) plates and screened for expressing strains by immunoblotting against HA-tag antibodies.

#### DIC imaging and ciliary/cellular phenotype analysis

For cell imaging, cells cultured in liquid medium were fixed with 5% glutaraldehyde solution followed by being placed on a slide with a coverslip and observed and captured by LAS V4.0 imaging software on a Leica DMI4000 B inverted microscope (Leica, Germany) with a 40 × or 63× objective (HCX PL FLUOTAR L 40×/0.60, 506203; HCX PL FLUOTAR L 63 ×/0.70, 506217) for differential interference contrast (DIC) images. In general, ciliary lengths from ∼50 cells or cell areas from ∼200 cells were measured by ImageJ (National Institutes of Health, USA). At least 200 cells were counted for cell numbers. All original measurement and counting data were statistically analyzed and plotted with GraphPad Prism (GraphPad Software, USA). All the experiments were conducted in triplicate.

#### Ciliary reassembly, disassembly, and elongation

Autolysin, the cell wall proteolytic enzyme produced during the *C. reinhardtii* cell mating process, was prepared following our previous protocol for the induced ciliation of palmelloid cells.^23^ The low-pH-indued deciliation and ciliary reassembly method is implemented to observe synchronized ciliary regeneration.^57^^,^^58^ The 20 mM sodium pyrophosphate (NaPPi) or 25 mM LiCl was added to the liquid M medium to induce ciliary disassembly or elongation over time, respectively.^49^^,^^59^ Cells were prepared for DIC imaging or immunoblot assays at all the indicated time points in the above cilia-related processes, as illustrated in the main text or figures. All the experiments were repeated three times.

#### Immunoblotting

In general, cell samples of ∼1 × 10^7^ cells at the indicated time points in the figures were collected by centrifugation for 1 min at 10,000 × *g*, immediately frozen in liquid nitrogen, and stored at −80°C until use. The frozen *C. reinhardtii* cell samples were then disintegrated via 50 μL of prechilled buffer A (50 mM Tris-HCl [pH 7.5], 10 mM MgCl_2_, 1 mM EDTA, 1 mM DTT) with cOmplete Mini EDTA-free Protease Inhibitor Cocktail (Cat. No. 11836170001, Roche, Switzerland), dispersed and then boiled in 1 × SDS sample buffer for 10 min, followed by SDS–PAGE and immunoblotting analysis.

The primary and secondary antibodies were diluted into 3% skim milk powder in TBST for use: anti-HA high affinity (clone 3F10, rat monoclonal IgG, 1:3000; Cat. No. 11867423001; RRID: AB_390918, Roche, Switzerland), anti-α-tubulin (rabbit polyclonal IgG, 1:5000; Cat. No. 11224-1-AP; RRID: AB_2210206, Proteintech, USA), anti-NPHP4 (rabbit polyclonal IgG, 1:5000; custom-made from ABclonal, China), anti-NPHP8 (rabbit polyclonal IgG, 1:5000; custom-made from ABclonal, China), anti-BBS1 (rabbit polyclonal IgG, 1:5000; custom-made from ABclonal, China), and HRP-conjugated goat anti-mouse, goat anti-rabbit and goat anti-rat IgG (1:5000; Cat. No. 115-035-003; RRID: AB_10015289, Cat. No. 111-035-003; RRID: AB_2313567, Cat. No. 112-035-003; RRID: AB_2338128, Jackson, USA). The corresponding specific immunoreactive bands were visualized and captured with an Amersham Imager 600 Control (GE, USA).

#### Immunostaining

Immunostaining was performed essentially as previously described.^57^ To locate the ciliary transition zone proteins NPHP4 and NPHP8, the indicated cells were resuspended in MT buffer (30 mM HEPES [pH 7.2], 3 mM EGTA, 1 mM MgSO_4_, 25 mM KCl), followed by fixation and permeabilization in 100% prechilled methanol for 20 min at −20°C. The samples were subsequently rehydrated in PBS, blocked with goat blocking buffer containing 5% goat serum (Cat. No. E674004, Cat. No. E510009, Sangon Biotech, China), and then incubated with primary antibodies at 4°C overnight, followed by incubation with secondary antibodies at 37°C for 1–2 h.

The following primary antibodies were diluted into the above goat-blocking buffer for use: anti-HA high affinity (clone 3F10, rat monoclonal IgG, 1:50; Cat. No. 11867423001; RRID: AB_390918, Roche, Switzerland), anti-acetylated α-tubulin (clone 6-11B-1, mouse monoclonal IgG, 1:200; Cat. No. T7451; RRID: AB_609894, Sigma–Aldrich, USA), anti-NPHP4 (rabbit polyclonal IgG, 1:500; custom-made from ABclonal, China), and anti-NPHP8 (rabbit polyclonal IgG, 1:500; custom-made from ABclonal, China). The secondary antibodies were diluted in secondary antibody dilution buffer containing 5% goat serum (Cat. No. E674005, Cat. No. E510009, Sangon Biotech, China) for use: goat anti-mouse IgG H&L (Alexa Fluor 594), goat anti-rat IgG H&L (Alexa Fluor 488), and goat anti-rabbit IgG H&L (Alexa Fluor 488) (1:500; Cat. No. ab150120; RRID: AB_2631447, Cat#ab150157; RRID: AB_2722511, Cat. No. ab150077; RRID: AB_2630356, Abcam, UK).

After three 5-min washes with PBST, one wash with PBS, and one wash with Milli-Q, the resulting samples on the slides were mounted with DAPI-Fluoromount-G (Cat. No. 0100-20, SouthernBiotech, USA), sealed with nail polish and air dried for at least 2 h before observation and imaging. Imaging was performed on a Leica TCS SP8 confocal laser microscope (Leica, Germany) equipped with a 63× oil immersion objective (HCX PL APO 63×/1.40–0.60 OIL CS, 506188). Raw image data were captured and processed in Leica Application Suite X (Leica, Germany), ImageJ (National Institutes of Health, USA), and Adobe Photoshop (Adobe Systems Inc., USA) and assembled via Adobe Illustrator (Adobe Systems Inc., USA).

### Quantification and statistical analysis

In general, all the experiments in the manuscript were independently repeated three times with similar results, and a representative experiment is presented. The number (*n*) of samples used for the measurements is indicated in the corresponding figure legends. All the data are presented as the mean ± SD with an error bar as indicated. All the statistical tests were performed in GraphPad Prism (version 9.0). One-way or two-way ANOVA with the recommended test was used to compare differences among multiple groups. Differences between various groups were considered significant if the *p* value was <0.05. No data were excluded from the analyses.
