# Supplementary material for: Profiling truncated variants of TCTN1 unveils the essential role of its integrity for ciliogenesis
Source: iScience. 2025 Nov 24;28(12):114190. doi: 10.1016/j.isci.2025.114190 (PMC12723291; doi:10.1016/j.isci.2025.114190)
Supplement: Document S1. Figures S1–S9 and Table S1 [file mmc1.pdf]

iScience, Volume *28*

Supplemental information

Profiling truncated variants of TCTN1 unveils the essential role of its integrity for ciliogenesis

Huimin Yu, Lin Jiang, Chuang Xu, Ya Li, Suhui Wang, Zhao Ma, Yunyao Qian, Yiqiong Wu, Chenyu Miao, Zhouzhou Dong, and Liang Wang

Supplemental information titles and legends

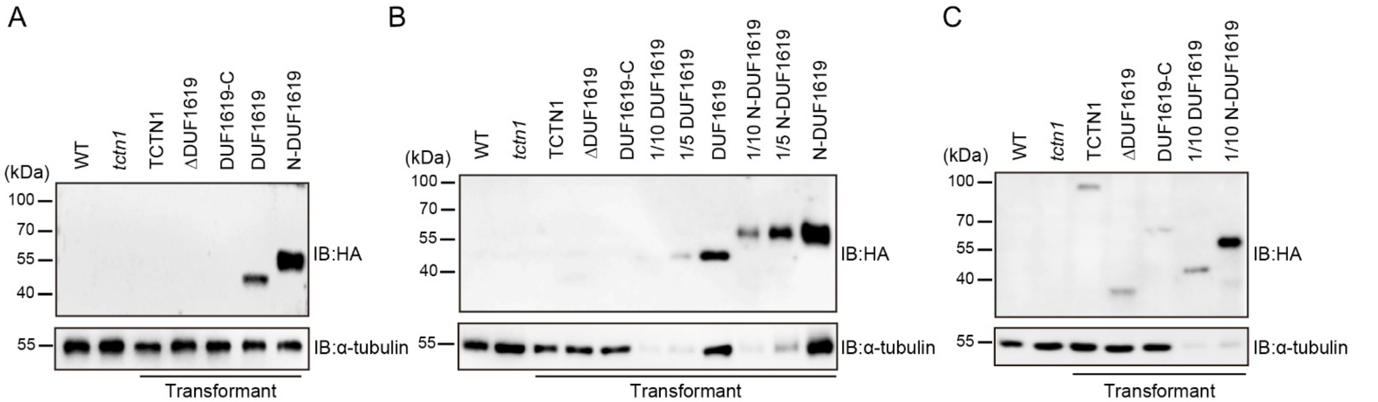

Figure S1. The differential levels of truncated TCTN1 in the corresponding transformants, Related to Figure 1.

(A-C) The transformants expressing the truncated versions of TCTN1 (DUF1619, ΔDUF1619, N-DUF1619, and DUF1619-C), as well as full-length TCTN1 (TCTN1), along with WT and *tctn1* cell lines, were analyzed by immunoblotting against the HA tag. WT and *tctn1* cell samples served as negative controls. α-tubulin was utilized as a loading control. Due to the high abundance of DUF1619 and N-DUF1619, reduced sample amounts (1/5 and/or 1/10 of the standard amount) were loaded for WB analysis, as shown in B and C. The molecular masses of standard proteins in kDa are indicated.

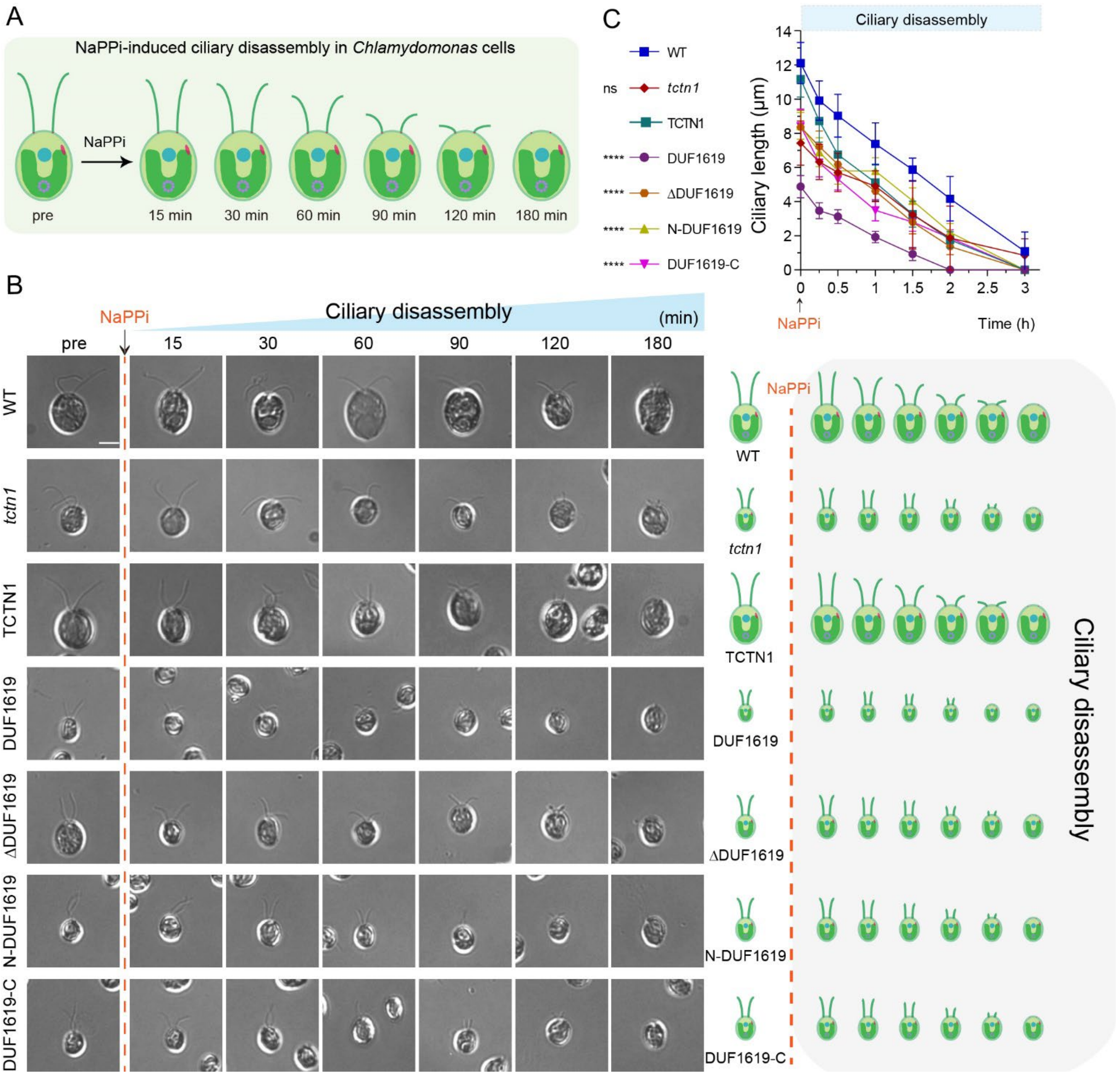

Figure S2. Truncated TCTN1 mutants display a shorter ciliary disassembly time, Related to Figure 3.

(A) Cartoon depicting the NaPPi-induced ciliary disassembly process in *Chlamydomonas* cells. (B) DIC images displaying NaPPi-induced ciliary disassembly in WT, *tctn1*, TCTN1, and truncated mutant cells. The black arrow indicates the time of NaPPi treatment. The sampling time is timed by adding NaPPi as the starting point. pre, before adding NaPPi. Scale bar, 5 μm. (C) Ciliary disassembly curves after NaPPi in WT, *tctn1*, TCTN1, and truncated mutant cells were plotted to view their differential disassembly kinetics. The data are presented as the means ± SDs (*n*=50). Statistical significance to the WT group was determined via two-way ANOVA. ns, not significant. ****, $P<0.0001$.

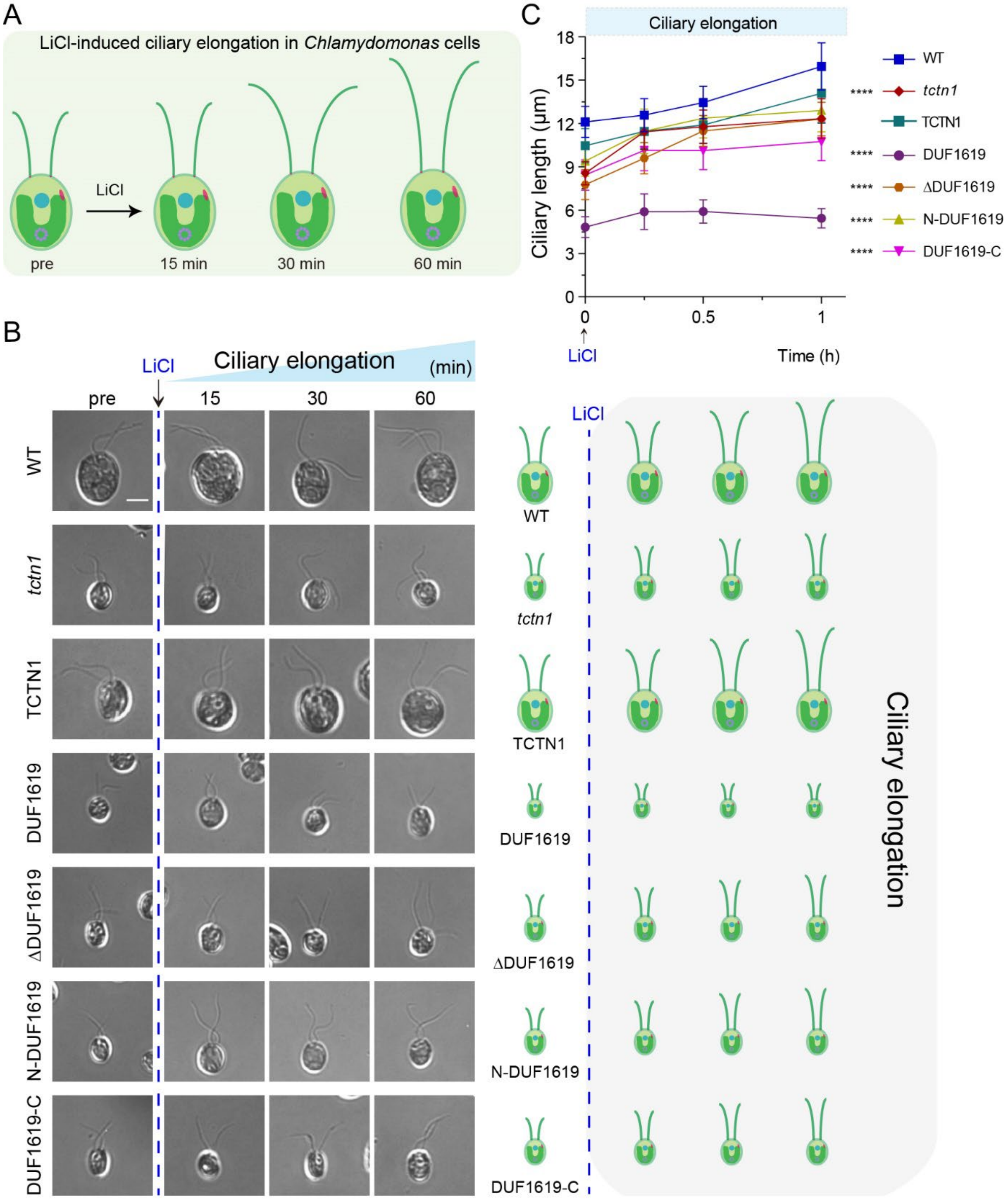

Figure S3. The TCTN1(DUF1619) cell line exhibits almost constant ciliary length under LiCl stimulus, Related to Figure 3.

(A) Cartoon depicting the LiCl-induced ciliary elongation process in *Chlamydomonas* cells. (B) DIC images displaying LiCl-induced ciliary elongation in WT, *tctn1*, TCTN1, and truncated mutant cells. The black arrow indicates the time of LiCl treatment. The sampling time is timed by adding LiCl as the starting point. pre, before adding LiCl. Scale bar, 5 μm. (C) Ciliary elongation curves after LiCl treatment in WT, *tctn1*, TCTN1, and truncated mutant cells were plotted to view their differential elongation kinetics. The data are presented as the means ± SDs (*n*=50). Statistical significance to the WT group was determined via two-way ANOVA. ****, *P*<0.0001.

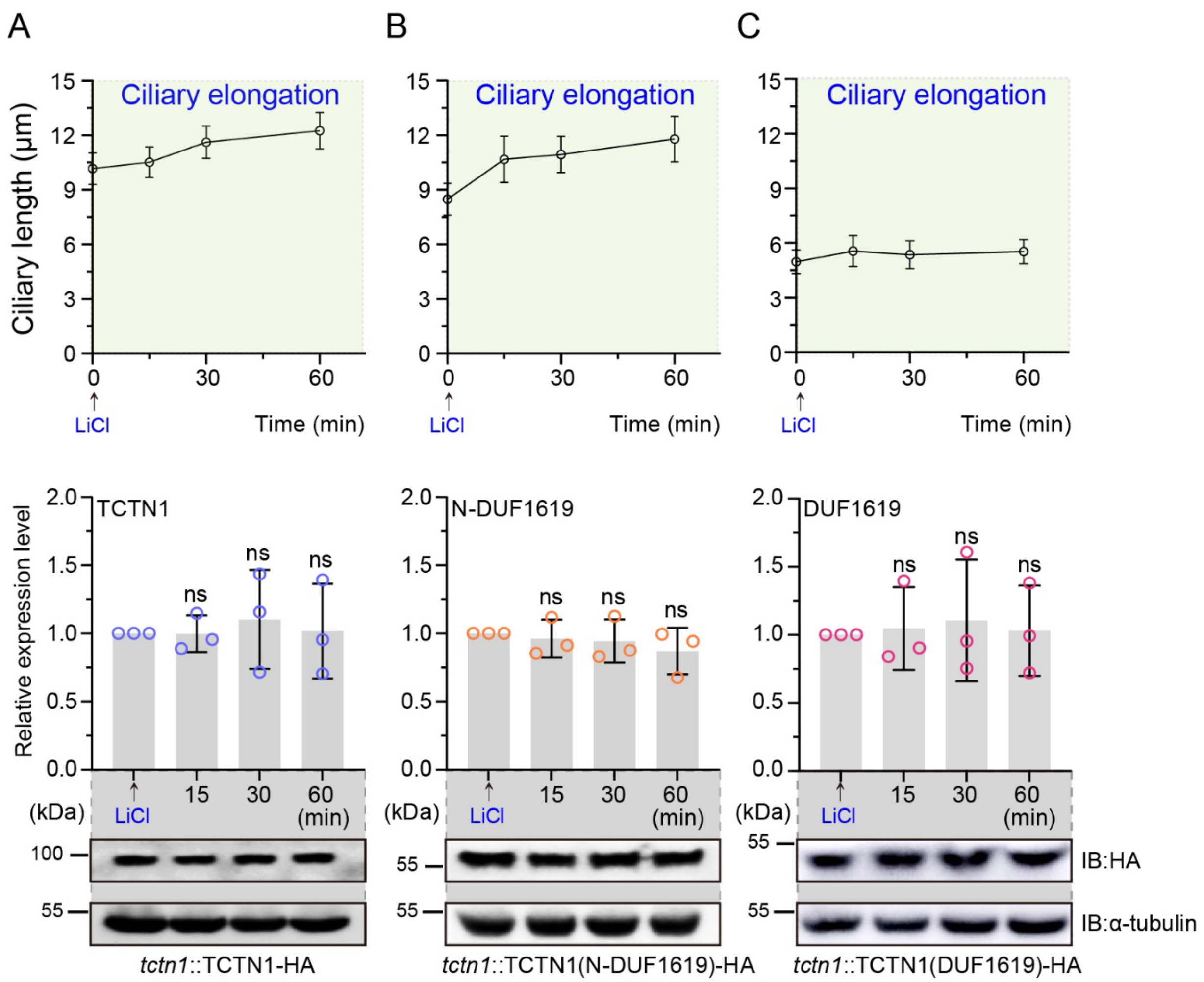

Figure S4. The C-terminus of TCTN1 is not needed for stabilizing TCTN1 underlying ciliary elongation, Related to Figure 4.

(A-C) Ciliary elongation graphs and the corresponding immunoblot analysis, along with band intensity by gray values in *tctn1*::TCTN1-HA (A), *tctn1*::TCTN1(N-DUF1619)-HA (B), and *tctn1*::TCTN1(DUF1619)-HA (C) cells, were plotted to view the changes in the corresponding protein levels. The data in the ciliary length curves are the means ± SDs (n=50). The data at the relative protein level are presented as the means ± SDs (n=3). Statistical significance to the time point 0 group was determined via one-way ANOVA. ns, not significant. The molecular masses of standard proteins in kDa are indicated. α-tubulin was used as a loading control.

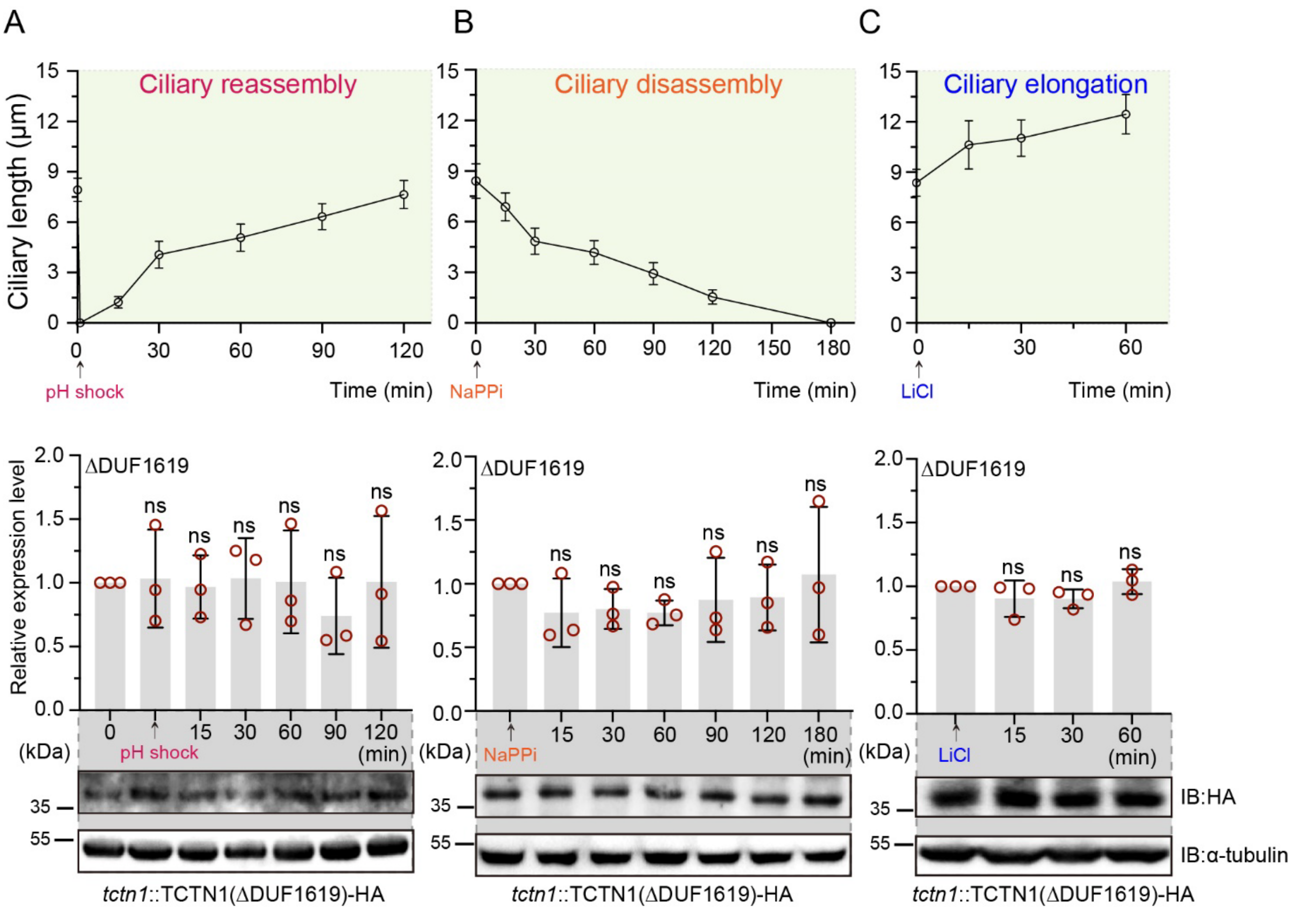

Figure S5. Profiling the protein level of TCTN1(ΔDUF1619) during ciliary reassembly, disassembly, and elongation, Related to Figure 4.

(A-C) Ciliary reassembly (A), disassembly (B), and elongation (C) graphs, along with the corresponding immunoblot analysis and band intensity by gray values, of *tctn1*::TCTN1(ΔDUF1619)-HA cells, were plotted to view the changes in the corresponding protein levels. The data in the ciliary length curves are the means ± SDs (*n*=50). The data at the relative protein level are presented as the means ± SDs (*n*=3). Statistical significance to the time point 0 group was determined via one-way ANOVA. ns, not significant. The molecular masses of standard proteins in kDa are indicated. α-tubulin was used as a loading control.

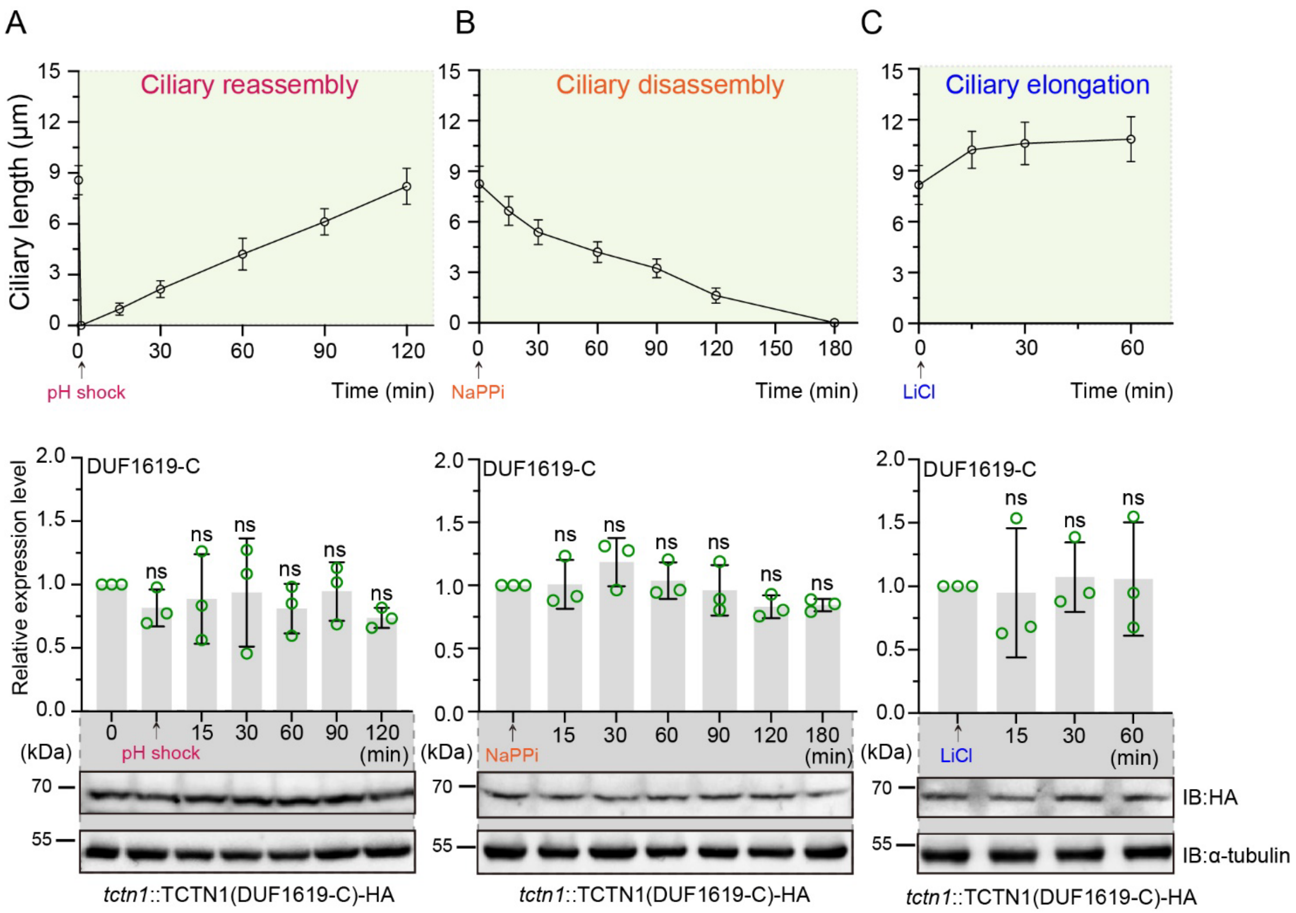

Figure S6. Profiling the protein level of TCTN1(DUF1619-C) during ciliary reassembly, disassembly, and elongation, Related to Figure 4.

(A-C) Ciliary reassembly (A), disassembly (B), and elongation (C) graphs, along with the corresponding immunoblot analysis and band intensity by gray values, of *tctn1*::TCTN1(DUF1619-C)-HA cells, were plotted to view the changes in the corresponding protein levels. The data in the ciliary length curves are the means ± SDs (n=50). The data at the relative protein level are presented as the means ± SDs (n=3). Statistical significance to the time point 0 group was determined via one-way ANOVA. ns, not significant. The molecular masses of standard proteins in kDa are indicated. α-tubulin was used as a loading control.

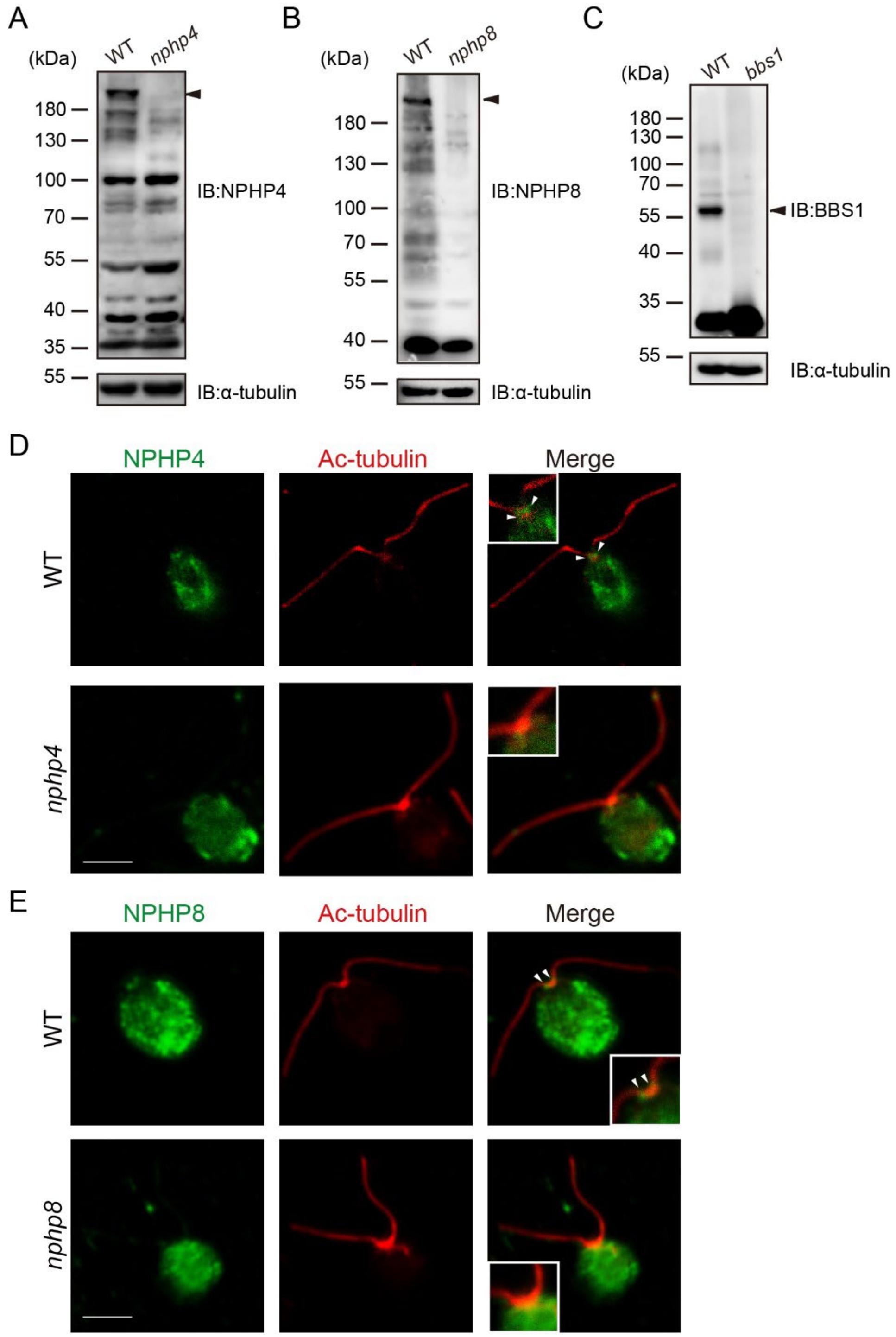

Figure S7. Verification of the specificity of the NPHP4, NPHP8, and BBS1 antibodies used in this study, Related to Figures 6, and 7.

(A-C) Immunoblot verification of the validation of custom-made antibodies against the ciliary proteins NPHP4 (A), NPHP8 (B), and BBS1 (C) in the WT and respective genetic mutants (*nphp4*, *nphp8*, and *bbs1*). The black arrowhead indicates the specific immunoreactive band only in the WT sample. The molecular masses of standard proteins in kDa are indicated. α-tubulin was used as a loading control. (D-E) Immunostaining verification of the antibodies against the ciliary transition zone proteins NPHP4 (D) and NPHP8 (E) in the WT and respective genetic mutants (*nphp4*, *nphp8*). The signal of acetylated α-tubulin (Ac-tubulin, red) marks the cilium. The white arrowheads

indicate the ciliary base (ciliary transition zone). The insets show higher magnification views of the transition zone region. Scale bar, 5 μm.

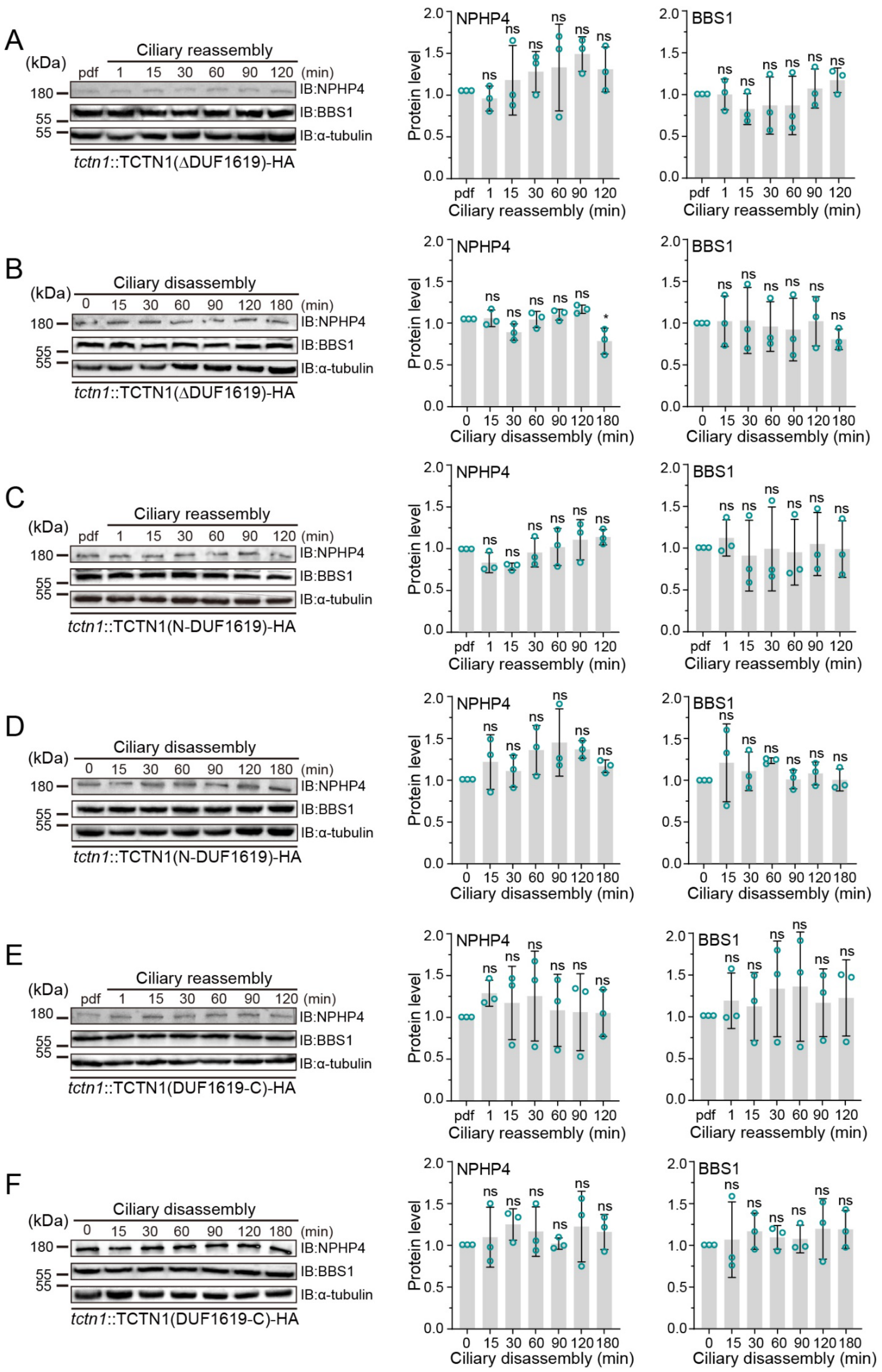
A
(kDa)
Ciliary reassembly
pdf 1 15 30 60 90 120 (min)
180
55
55
IB:NPHP4
IB:BBS1
IB:α-tubulin
tctn1::TCTN1(ΔDUF1619)-HA
NPHP4
BBS1
Protein level
ns
Ciliary reassembly (min)
B
Ciliary disassembly
0 15 30 60 90 120 180 (min)
tctn1::TCTN1(ΔDUF1619)-HA
*
Ciliary disassembly (min)
C
tctn1::TCTN1(N-DUF1619)-HA
D
tctn1::TCTN1(N-DUF1619)-HA
E
tctn1::TCTN1(DUF1619-C)-HA
F
tctn1::TCTN1(DUF1619-C)-HA

Figure S8. The levels of NPHP4 and BBS1 during ciliary reassembly and disassembly are constant in truncated TCTN1 mutants, Related to Figure 6.

(A-B) Immunoblot and gray value profiling for changes in the levels of ciliary proteins (NPHP4 and BBS1) during ciliary reassembly (A) and disassembly (B) events in *tctn1*::TCTN1(ΔDUF1619)-HA. (C-D) Immunoblot and gray value profiling for changes in the levels of ciliary proteins (NPHP4 and BBS1) during ciliary reassembly (C) and disassembly (D) events in *tctn1*::TCTN1(N-DUF1619)-HA. (E-F) Immunoblot and gray value profiling for changes in the levels of ciliary proteins (NPHP4 and BBS1) during ciliary reassembly (E) and disassembly (F) events in *tctn1*::TCTN1(DUF1619-C)-HA. The data at the relative protein level are presented as the means ± SDs (n=3). Statistical significance to the time point 0 or pdf group was determined via one-way ANOVA. ns, not significant. *, P<0.1. pdf, predeflagellated. The sampling times are as indicated in this figure. The molecular masses of standard proteins in kDa are indicated. α-tubulin was used as a loading control.

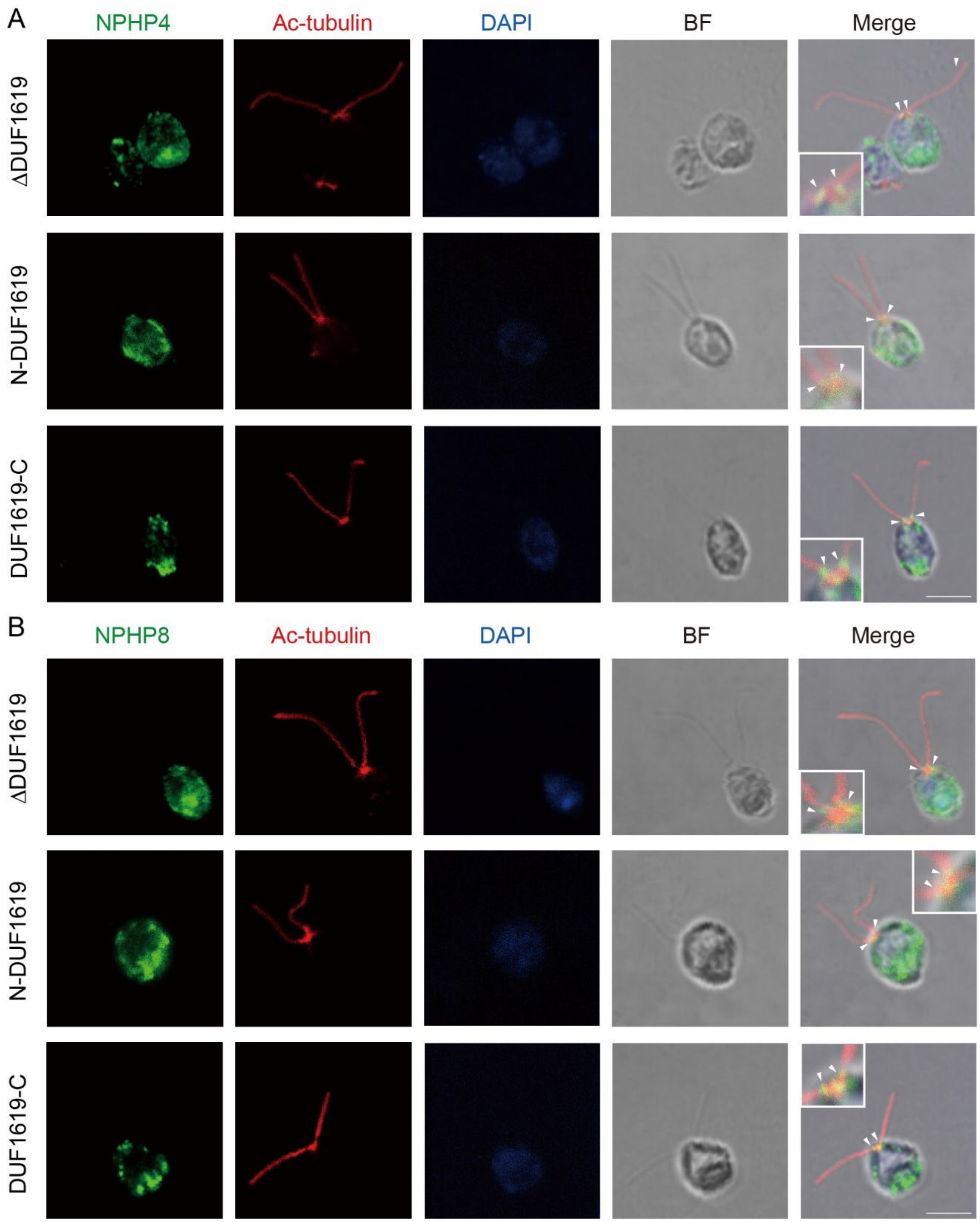

Figure S9. Ciliary transition zone molecules NPHP4 or NPHP8 still localize to the base puncta of cilia in truncated TCTN1 mutants, Related to Figure 7.

(A-B) Immunostaining images showing the ciliary base puncta of NPHP4 (A) or NPHP8 (B) in *tctn1*::TCTN1(ΔDUF1619)-HA (ΔDUF1619), *tctn1*::TCTN1(N-DUF1619)-HA (N-DUF1619), and *tctn1*::TCTN1(DUF1619-C)-HA (DUF1619-C) cells. All the cells were immunostained with anti-NPHP4 (green) or anti-NPHP8 (green), and anti-acetylated α-tubulin (Ac-tubulin, red) antibodies. The nuclei were stained with DAPI (blue). The white arrowheads indicate the ciliary base (ciliary transition zone). The insets show higher magnification views of the transition zone region. The brightfield (BF) and merge channels are also showed. Scale bar, 5 μm.

Table S1. Primers for constructions of truncated *TCTN1*-expressing plasmids, Related to STAR Methods

Primers	Sequence 5' to 3'
DJ-F	GACCATGATTACGAATTCGATATCACAATGTCCGACACCACTCTGTC
DJ-1-R1	GTTCGCCGTGAGGTTGGTGCTGGTTGG
DJ-1-F1	GCACCAACCTCACGGCGAACTTGTTGGTGAGCGGGCAGTCCGATC
DJ-1-R	CAGGCCTCCCGCGGCCGCGATATCCGCACGCGCACGGAATGGCAA AAG
DJ-B-R1	CCTGGCAAGCTTACTGAAGCTTAACC
DJ-B-F1	CTTCAGTAAGCTTGCCAGGATGTGCAACTGTGACCTCCGGCAAGAG
DJ-B-R	GACAGGCCTCCCGCGGCCGCGATATCTGGGAAACCGGTGACGTAG CCGGGG
I124-F2	ATGCCCAGGATGGGCACATC
I124-R1	ACCGCATCGATGTATCCG
DJ-SEQ-F1	CACACCGGCTGCAGGCAATGCG
DJ-SEQ-F2	CGCCGAGCAAGGTGCCCAGG
DJ-SEQ-F3	GCCATTGCACTCGTTCGGGC
DJ-SEQ-F4	CTCACCGCGAACCAGCTGCGTG
DJ-SEQ-F5	CAGCGCTTGAGGGCCGCTAC
DJ-SEQ-F6	GTGGGCGACAACCAGGAGCTG
DJ-SEQ-F7	GCATCTGAAGCATAGCCGCG
DJ-SEQ-F	CACTCATTAGGCACCCCAGGC
